# Retinal changes detected by diffuse reflectance spectroscopy in parkinsonian monkeys

**DOI:** 10.1117/1.NPh.12.2.025008

**Published:** 2025-05-05

**Authors:** Jonathan Munro, Elahe Parham, Damon DePaoli, Nicolas Lapointe, Cleophace Akitegetse, Shirley Fecteau, Dominic Sauvageau, Thérèse Di Paolo, Daniel C. Côté, Martin Parent

**Affiliations:** a Université Laval, CERVO Brain Research Center, Quebec City, Quebec, Canada; b Zilia Inc, Quebec City, Quebec, Canada; c Université Laval, CHU de Québec Research Center, Quebec City, Quebec, Canada

**Keywords:** biomarkers, MPTP, neurodegenerative disease, nonhuman primates, Parkinson’s disease, retina

## Abstract

**Significance:**

Parkinson’s disease (PD) is diagnosed when 50% neurodegeneration has occurred. The retina could provide biomarkers that would allow for earlier diagnosis. Retinal spectroscopy is a technique that could be used to find such biomarkers.

**Aim:**

We aimed to find new diagnostic biomarkers for PD following detailed spectral examinations of the retina.

**Approach:**

The newly developed Zilia Ocular device was used to perform spectrometric scans of the optic nerve head (ONH) and the retina of four cynomolgus monkeys (*Macaca fascicularis*) before and after the administration of 1-methyl-4-phenyl-1,2,3,6-tetrahydropyridine (MPTP), a neurotoxin used to produce the gold-standard animal model of PD. From the spectrometric data, the blood oximetry was calculated, and the diffuse reflectance spectra (DRS) were analyzed to find variations between the two experimental conditions. Post-mortem analyses were also performed on the retina of the four parkinsonian monkeys and four additional control animals.

**Results:**

The analysis of the DRS indicated a lower slope between the 480- and 525-nm wavelengths in both the ONH and the retina. Post-mortem measurements of the retinal layer thicknesses showed that the outer nuclear layer was significantly thinner in MPTP-intoxicated monkeys, compared with controls. Altogether, these results indicate that MPTP altered the optical properties of the ONH and the retina and show that these variations might be explained by MPTP-induced structural changes in the eye fundus, as observed post-mortem.

**Conclusions:**

Overall, our results indicate that spectroscopy could be used as a noninvasive method to detect changes in the retina that occur in PD and that such changes could represent retinal biomarkers for improved diagnosis.

## 1 Introduction

Parkinson’s disease (PD) is characterized by the progressive loss of dopamine (DA) neurons of the substantia nigra pars compacta (SNc), leading to the appearance of debilitating motor symptoms, in addition to nonmotor symptoms.^1^^,^^2^ This neurodegenerative disorder is incurable, and although some motor symptoms can be alleviated by the administration of the DA metabolic precursor levodopa, such pharmacotherapy eventually leads to adverse side effects in the form of dyskinesias, leaving very limited options for alternative treatments.^3^^–^^6^ As the second most common neurodegenerative disease next to Alzheimer’s disease (AD), it is a burden on healthcare services worldwide, especially with the rapidly increasing aging population.^7^ For this reason, research efforts are being made to find alternative ways to detect PD, alleviate its symptoms, slow its progression, and find a cure.

PD is diagnosed by clinical observation of motor symptoms by a neurologist. However, by the time motor symptoms are expressed, around 50% of DA neurons in the SNc have been lost, meaning that significant neurodegeneration has already occurred.^8^ This limits the ability to conduct research on treatments that could stop or slow the progression of the disease. Many neuroprotective treatments have already been investigated, but until it is possible to administer such treatments to individuals with PD prior to the onset of their motor symptoms and significant neurodegeneration, it is very difficult to measure their effectiveness at delaying disease progression. Therefore, it is imperative to improve the diagnostic method to successfully apply neuroprotective treatments during the prodromal stage of the disease.

One potential investigative source of PD biomarkers for early diagnosis is the retina. As a part of the central nervous system, it is possible to have its structure and activity monitored using noninvasive techniques. As such, it has become a focused target of research investigating whether detectible retinal alterations could reflect changes occurring in the brain that characterize neurodegenerative diseases. Vision problems, such as contrast sensitivity and visual acuity, are known nonmotor symptoms of PD, and patients often report them before diagnosis, suggesting that changes could be occurring to the retina prior to the onset of motor symptoms.^9^^,^^10^ In addition, some morphological and physiological changes have been detected in the retina of PD patients.^11^^–^^16^ Such changes might represent biomarkers that could contribute to early diagnosis of PD.

Ocular oximetry is a noninvasive recording technique, which quantifies blood oxygen saturation in structures of the eye fundus. By detecting variances in light absorption from acquired spectra, it is possible to calculate differences between oxygenated and deoxygenated hemoglobin in large blood vessels^17^^,^^18^ and tissues of the eye fundus.^19^^,^^20^ Abnormal oxygen metabolism and vascular morphology are known pathophysiological features of some neurodegenerative diseases, including PD.^21^^–^^25^ Therefore, oxygen saturation presents itself as a potential biomarker for PD with ocular oximetry being an effective noninvasive method to detect it.

Despite its promising implications, there have been few investigations into the use of ocular oximetry as a diagnostic tool. Previous studies have found links between altered blood oxygen content with various eye disorders, such as diabetic retinopathy, macular degeneration, and glaucoma, and Alzheimer’s disease and multiple sclerosis (MS).^22^^,^^23^^,^^26^^–^^28^ However, the tools used to conduct these studies perform oximetry in the major blood vessels, without the ability to target specific structures, such as the optic nerve head (ONH) and the macula, which may be differentially affected by the disease at earlier stages.^29^ Furthermore, these studies were conducted as case-control, which does not account for potential inter-individual variability. A new device has recently been developed that enables the acquisition of diffuse reflectance spectra (DRS) from specific locations in the eye fundus, via a technique called targeted spectroscopy. Besides calculating oximetry, acquired spectra can be further analyzed to obtain additional information on potential physiological or morphological changes in the eye fundus.

The aim of this study was to use DRS to find potential retinal or ocular biomarkers to improve the diagnosis of PD. Spectra were obtained from specific locations in the retina and ONH of nonhuman primates (NHP), before and after the administration of 1-methyl-4-phenyl-1,2,3,6-tetrahydropyridine (MPTP), a neurotoxin used to produce the gold-standard animal model of PD. Blood oxygen saturation was calculated at each region, and the recorded spectra were analyzed to determine MPTP-induced changes within subjects. Finally, post-mortem analysis of the retina was performed to find potential associations between spectral signatures and morphological and neurochemical changes in the NHP eye fundus.

## 2 Methods

### 2.1 Animals

This study was conducted using four ovariectomized cynomolgus monkeys (*Macaca fascicularis*), all aged 5 years old and weighing between 2.9 and 3.5 kg (Table 1). Ovariectomy was performed before MPTP administration to model PD that affects aging menopausal women having a reduced estrogen level known to have a neuroprotective effect on DA neurons. Animals were housed under a 12-h light-dark cycle with unlimited access to food and water. Experiments were approved by the Comité de Protection des Animaux de l’Université Laval, in accordance with the Canadian Council on Animal Care’s guide to the Care and Use of Experimental Animals. The minimum necessary number of animals was used to complete this study.

**Table 1:** Information on MPTP-intoxicated and control NHPs.

Animal ID	Group	Age (years)	Sex	Weight (kg)	Motor disability score (/16)^a^	Total MPTP (mg)
CTL1	Control	3	Male	2.9	NA	NA
CTL2	Control	2	Male	2.9	NA	NA
CTL3	Control	2	Male	2.5	NA	NA
CTL4	Control	2	Male	2.5	NA	NA
MPTP1	MPTP	5	Female	3.15	9.41	14.11
MPTP2	MPTP	5	Female	3.25	9.37	9.46
MPTP3	MPTP	5	Female	3.15	6.72	9.91
MPTP4	MPTP	5	Female	3.55	7.85	12.67

a Motor disability was assessed using the scale outlined by Hadj Tahar et al.^30^

### 2.2 *In Vivo* Procedure and Timeline

Prior to the procedure, animals were lightly anesthetized using ketamine (10  mg/kg) with dexmedetomidine (0.4  mg/kg). Additional dosages were made at regular intervals during the procedure to prolong the anesthesia as needed. Isoflurane was not used as it can interfere with ocular recordings. Efforts were made to minimize the duration that the animals were under anesthesia.

**Fig. 1:**
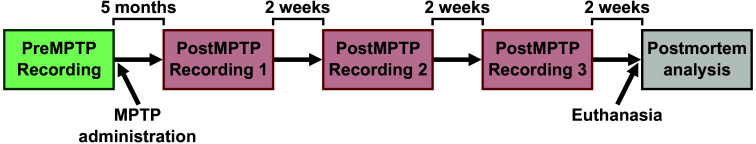
Experimental timeline. Each recording session occurred over 2 days with half a day devoted for each NHP. Post-mortem analyses were performed on a single retina from each animal and compared with a single retina from each of the four control NHPs.

The four monkeys went through two experimental blocks with each block being composed of three recording sessions. MPTP was administered immediately after the first block of recording sessions. The second block began 5 months following MPTP administration, when motor disabilities induced by the neurotoxin were considered stable, as the animal showed no behavioral sign of functional recovery. During each block, recording sessions were separated by 2-week intervals. Each recording session occurred over 2 full days, with half of each day being devoted to one of the four NHPs (Fig. 1). The recording protocol had to be adjusted during the first two preMPTP recording sessions, with the third recording session onward being conducted using the chosen method for best reproducibility. Therefore, preMPTP data were analyzed from the third recording session only.

**Fig. 2:**
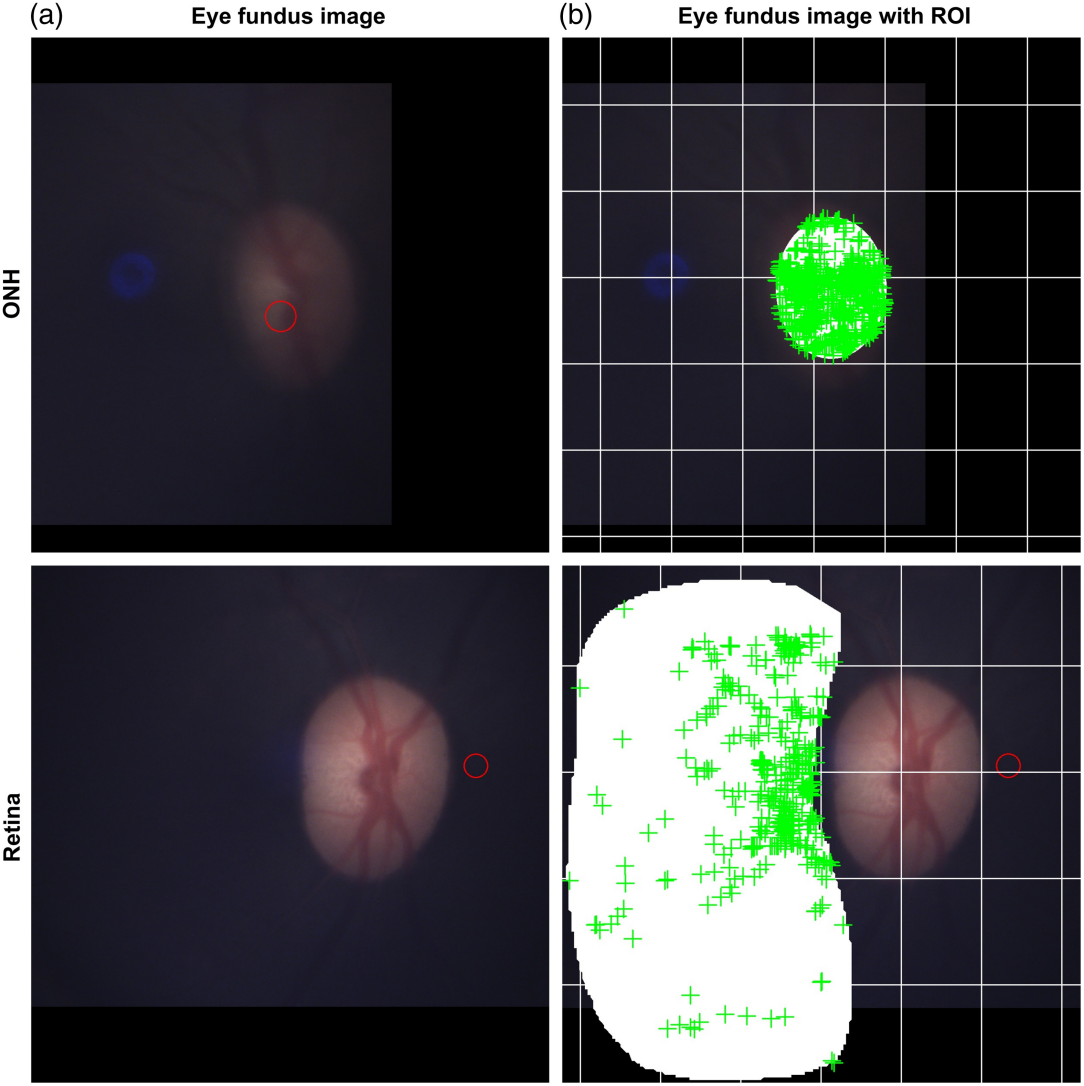
Reference images of the eye fundus used for region of interest (ROI) selection within the data acquisition software. (a) References of the eye fundus used by the software displaying the ONH in the correct relative position. (b) The same reference images but with the corresponding ROIs drawn in white for the ONH and retina regions and the green plus signs indicating each spectrum acquisition site. The red circles represent the location of the ROSA as indicated by the device during the acquisition.

The recordings made during the procedure were performed with the Zilia Ocular. This device is capable of performing targeted DRS by simultaneously imaging the eye fundus (23 deg) while recording spectra of reflected light from a user-targeted location (1.5 deg)^20^ (Fig. 2). A digital circle overlay on the fundus image precisely identifies the region of spectral acquisition (ROSA) on the retina. Both the ROSA and recorded spectra were visualized in real time allowing for accurate recordings and for adjusting the acquisition parameters, such as light intensity, to account for differences among recording regions in light absorption, ensuring sufficient signal strength and preventing over-saturation. Although originally designed to be used in humans, for this specific study, a custom-made chin and face mount was implemented to allow for its use with NHPs.

To begin the procedure, the pupils were dilated with Tropicamide (1%) and Phenylephrine (2.5%). The lightly anesthetized monkey was placed in a sphinx position, its chin lying on the mount. Recordings were made from each eye, one at a time, with the recorded eye being held open using an eye speculum and the other eye kept shut to prevent drying. Moisturizing eye drops were regularly applied to the open eye (Systane Gel Drops, Alcon, Fort Worth, Texas, United States). The position of the device was adjusted until the eye fundus was clearly visible on the device screen. Separate recordings were made at two regions of interest (ROIs): within the area of the ONH, the retinal tissue surrounding the ONH. During the acquisition, light intensity was adjusted to obtain nonsaturated spectra with sufficient signal intensity. Light emitting diode (LED) intensities were 0.1224 mW within the ONH and 0.6936 mW within the retina.

### 2.3 MPTP Administration

Following the third recording session, MPTP (product no. M0896, Sigma, Kawasaki, Japan) was delivered at a concentration of 8.61  mg/mL via a subcutaneous osmotic mini-pump continuously for 14 days. Additional intramuscular MPTP was injected as required until stable parkinsonian symptoms had been established. The mean total MPTP delivered was 11.54±2.23  mg (Table 1). The scoring of parkinsonian symptoms was made using a scale developed specifically for NHPs, which assesses the behavioral response to MPTP for posture, mobility, climbing, gait, grooming, vocalization, social interaction, and tremor.^30^ Assessments were made every 15 min during a 2-h period on 2 consecutive days, the week prior to the postMPTP block of recording sessions. The four animals scored between 6.72 and 9.41 out of 16.00 (mean of 8.34±1.30), indicating moderate to severe parkinsonian symptoms (Table 1). Their behavior remained stable for the rest of the experiment.

### 2.4 Euthanasia

The animals were euthanized 2 weeks following the sixth recording session. Transcardial perfusion was performed with 500 mL of phosphate-buffered saline (PBS, 0.1 M, pH 7.4) followed by 1.5 L of paraformaldehyde 4% (PFA) with glutaraldehyde 0.2% and finally 1 L of PFA 4%. Each of these solutions was kept on ice during delivery. Once complete, both eyes were removed, and an incision was made on the cornea before being placed in 4% PFA for 7 days.

### 2.5 Retinal Dissection and Preparation

Post-mortem analyses were performed on the retina of the four MPTP-intoxicated NHPs in addition to the retina of four control (CTL) NHPs (Table 1). The right eyes, used in the present study, were kept in a solution of sucrose (30%) with sodium azide (0.1%) for cryoprotection.

After cryoprotection, the retinas were dissected from the eyes. The cornea and optic lens were removed first. The eye orientation was determined using the optic nerve as a landmark. Four equally spaced cuts were made from the opening of the cornea toward the macula so that the retina could be spread flat with four “wings” at dorsal, ventral, medial, and lateral positions with the macula and ONH at the center. Using a brush, the retina with the epithelial layer was gently detached from the sclera until completely separated. The four wings were trimmed to create flat edges before each was divided into three equal quadrilateral sections. A cut was made on the left corner of the most distally located edge from the macula for orientation. The sections were then placed into individual wells containing the sucrose solution and stored at 4°C until use.

The most proximal sections to the macula within the medial wing were selected for analysis. The epithelial layer was removed from the retina as it was found to interfere with the visualization of the retinal layers. These were sliced perpendicular to the macula into 14  μm-thick transverse sections with a cryostat at −20°C and collected on microscope slides.

### 2.6 Immunohistochemistry and Retinal Thickness Measurements

To measure MPTP-induced structural differences in the retina, immunostaining was performed for the proteins THY1 (1/800, product no. AB181469, Abcam, Cambridge, United Kingdom) and 4,6-diamidino-2-phenylindole (DAPI) (product no. D-1388, Sigma). Microscope slides containing the retina sections were initially washed in a bath of PBS three times for 5 min each, before spending an hour incubating in a blocking solution of PBS with 2% normal donkey serum and 0.1% Triton X. THY1 was diluted in the same blocking solution, in which the slides were incubated overnight at 4°C in the dark. The next day, the sections were washed in PBS before incubating for 2 h in the secondary antibody 488 anti-mouse (1/400, product no. 711-545-152, Jackson, West Grove, Pennsylvania, United States) diluted in the blocking solution. After another PBS wash, the sections were covered in DAPI solution for 10 min before a final PBS wash. The slides were then coverslipped using Dako fluorescent mounting medium (product no. 53023, Agilent, Santa Clara, California, United States).

TIFF images were acquired of each retinal section from each animal using a slidescanner fluorescent imaging system (TISSUEscopeTM 4000, Huron Technologies, Waterloo, Ontario, Canada) and loaded into the ImageJ software. The metadata of the images provided their dimensions in micrometers, which we could use to calibrate the scale in ImageJ. Using the straight-line drawing tool, contours were drawn around each region of interest representing each layer of the retina. The area and perimeter measurements were recorded and used to calculate the width and length of an equivalent rectangle. The width of this rectangle was used as the thickness of the retinal layer. The regions of interest measured within the retina were the retinal nerve fiber layer (RNFL), the ganglion cell layer and inner plexiform layer (GCL + IPL), the IPL, the inner nuclear layer (INL), the outer plexiform layer (OPL), the outer nuclear layer (ONL), and the photoreceptor cell layer (PRCL). The entire thickness of each retina was also measured.

### 2.7 *In Vivo* Data Analysis

The Zilia Ocular recorded images of the eye fundus and the location of the ROSA during spectral acquisition. Continuous acquisitions were made at 2.67 Hz throughout the targeted regions. Within each eye, an average of 118.6±66.0 acquisitions was made in the ONH and 25.4±29.8 in the retina during an individual recording session. The data were filtered to remove spectra with either noisy signal or acquired from a blurry image. Image blurriness was attributed to eye dryness or movement, which would affect the localization of the measurement. Spectra were then separated into different eye fundus regions based on where they were acquired, namely, the ONH and the retinal region located temporal to the ONH, extending until the perifovea region of the macula. Blood oxygen saturation (StO2) was calculated from the recorded spectra data using the method described by Akitegetse et al.^19^^,^^31^ Given the distinct contrast between the dark-colored retina against the bright ONH observed in each image, this visual landmark was used for the alignment of all images. A custom-coded Python script with the OpenCV (Open Computer Vision) library was implemented to automate this process.

During a single recording session, data were obtained for both eyes of a given monkey. Blood oxygen saturation values from a region of interest were averaged for each session (Tables S1 and S3 in the Supplementary Material) and across baseline and MPTP sessions (Tables S2 and S4 in the Supplementary Material). Comparisons were first made within subjects, between the left and right eyes and between recording sessions of each condition (baseline and MPTP) to assess intra-individual variability. Paired comparisons were then made between baseline and MPTP conditions by averaging the blood oxygen saturation across all sessions for each condition. This was performed first within subjects and then averaged across all animals.

In addition to blood oxygen saturation, the natural logarithm of the DRS was analyzed to determine if any differences emerged following MPTP administration. DRS intensity values were obtained for every fifth wavelength between 480 and 620 nm. Each DRS was normalized using the absolute deviation to easily visualize differences in the spectra between experimental conditions. For each monkey, all spectra obtained for a given condition at a given location were then averaged. A difference was noted in the overall slope of the spectral shapes between baseline and MPTP conditions, with the greatest difference being between 480 and 525 nm. Therefore, from the averaged spectra, the slopes were calculated using the linear regression of the values between these wavelengths (Tables S5–S8 in the Supplementary Material).

### 2.8 Statistical Analysis

Within-subject comparisons for the *in vivo* data were performed using paired, nonparametric t-tests (Wilcoxon signed-rank test). Comparisons between the control and MPTP groups for the post-mortem analysis were performed using unpaired, nonparametric t-tests (Mann–Whitney test). Throughout the text, mean and standard deviation are used as central tendency and dispersion measures. A P<0.05 was required for the results to be considered statistically significant.

## 3 Results

### 3.1 Assessment of Intra-Individual Variability

No significant differences in blood oxygen saturation were observed between eyes and across recording sessions of a given animal. Therefore, further analysis was made by averaging data across eyes and recording sessions of a given animal (Tables S1–S4 in the Supplementary Material).

### 3.2 Blood Oxygen Saturation

**Fig. 3:**
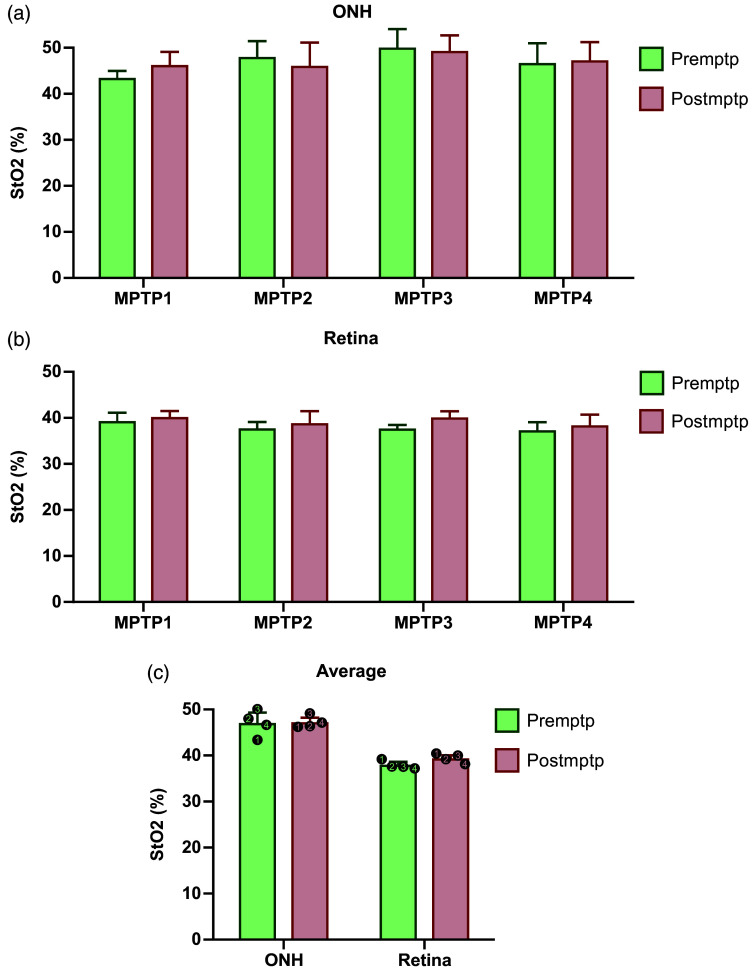
Comparison of the blood oxygen saturation (%) in the ONH (a) and the retina (b) between pre and postMPTP conditions for each animal. Each bar represents the mean and SD of the blood oxygen saturation from all acquisitions. (c) Comparison of the pre and postMPTP blood oxygen saturation in each region. The mean and SD were calculated from the average of the four animals.

Within the ONH, the recordings showed an average postMPTP change in blood oxygen saturation of +0.42% across all animals. Individually, the postMPTP change in blood oxygen saturation was +6.48% in MPTP1, −3.36% in MPTP2, −1.82% in MPTP3, and +1.06% in MPTP4 [Fig. 3(a)].

In the retinal region located temporal to the ONH, the recordings showed an average postMPTP change in blood oxygen saturation of +4.03% across all animals. Individually, the postMPTP change in blood oxygen saturation was +3.10% in MPTP1, +4.18% in MPTP2, +6.41% in MPTP3, and +2.49% in MPTP4 [Fig. 3(b)].

### 3.3 Spectra Slope Analysis

**Fig. 4:**
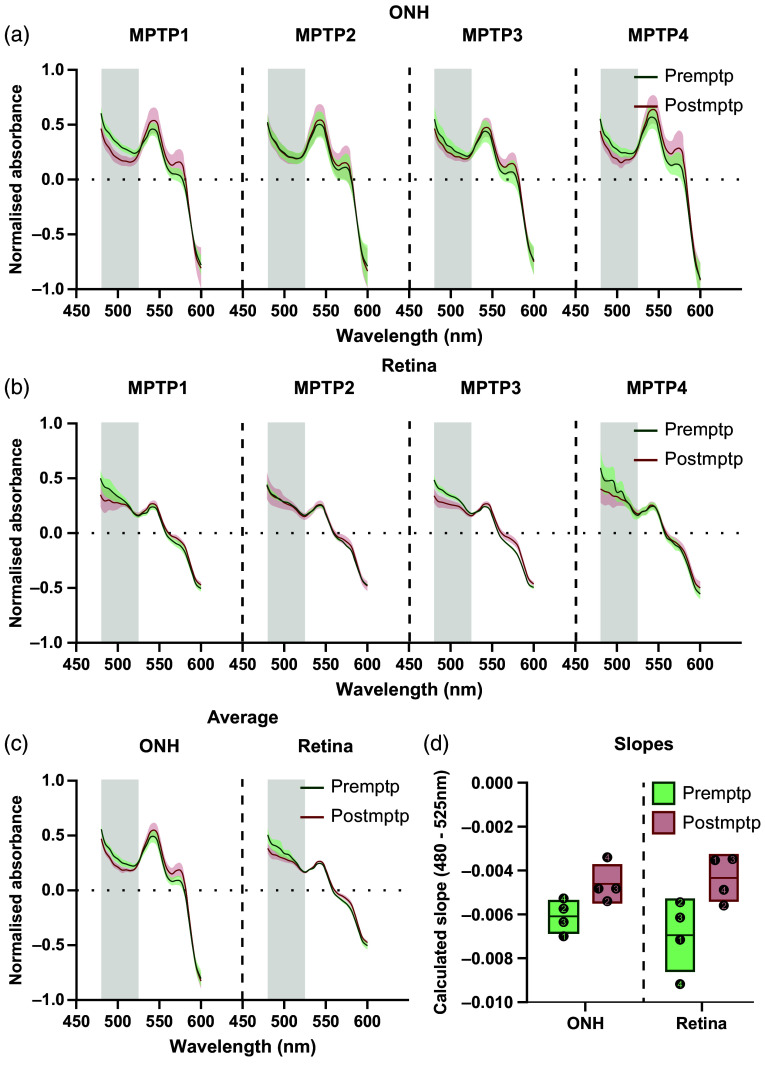
Comparison of absorbance spectra shapes in the ONH (a) and retina (b) between pre and postMPTP conditions for each animal. Normalization of each acquisition’s absorbance spectra was performed by calculating the absolute deviation for each wavelength. The mean (solid line) and SD (colored background) were then calculated from the normalized absorbance of each wavelength. The mean and SD were then calculated across the four animals (c) for each region. The gray background regions (a)–(c) represent the range of wavelengths (480 to 525nm) from which the slopes were calculated. (d) Comparison of the calculated slopes. The box plots represent the mean and standard deviation. Each numbered dot represents the calculated slope of an individual animal with numbers representing the NHP numbers, as reflected in Table 1.

Analysis of the normalized spectra before and after the administration of MPTP for each animal found that the biggest difference in spectral shape occurred at the 480 to 525 nm spectrum range. To quantify this difference, the slope of the spectra within this spectral range was calculated as the average for all pre- and postMPTP sessions within each animal. Within the ONH, the recordings showed an average postMPTP change in slope of −24.30% across all animals (−0.00610±0.00075 for preMPTP versus −0.00460±0.00085 for postMPTP). Individually, the postMPTP change in slope was −31.08% for MPTP1, −5.96% for MPTP2, −24.20% for MPTP3, and −35.40% for MPTP4 (Fig. 4).

Within the retinal region, the recordings showed an average postMPTP change in slope of −37.53% (−0.0069±0.0016 for preMPTP versus −0.0043±0.0010 for postMPTP). Individually, the postMPTP change in slope was −50.87% for MPTP1, +2.83% for MPTP2, −43.49% for MPTP3, and −47.05% for MPTP4 (Fig. 4).

### 3.4 Post-mortem Data Analysis

**Fig. 5:**
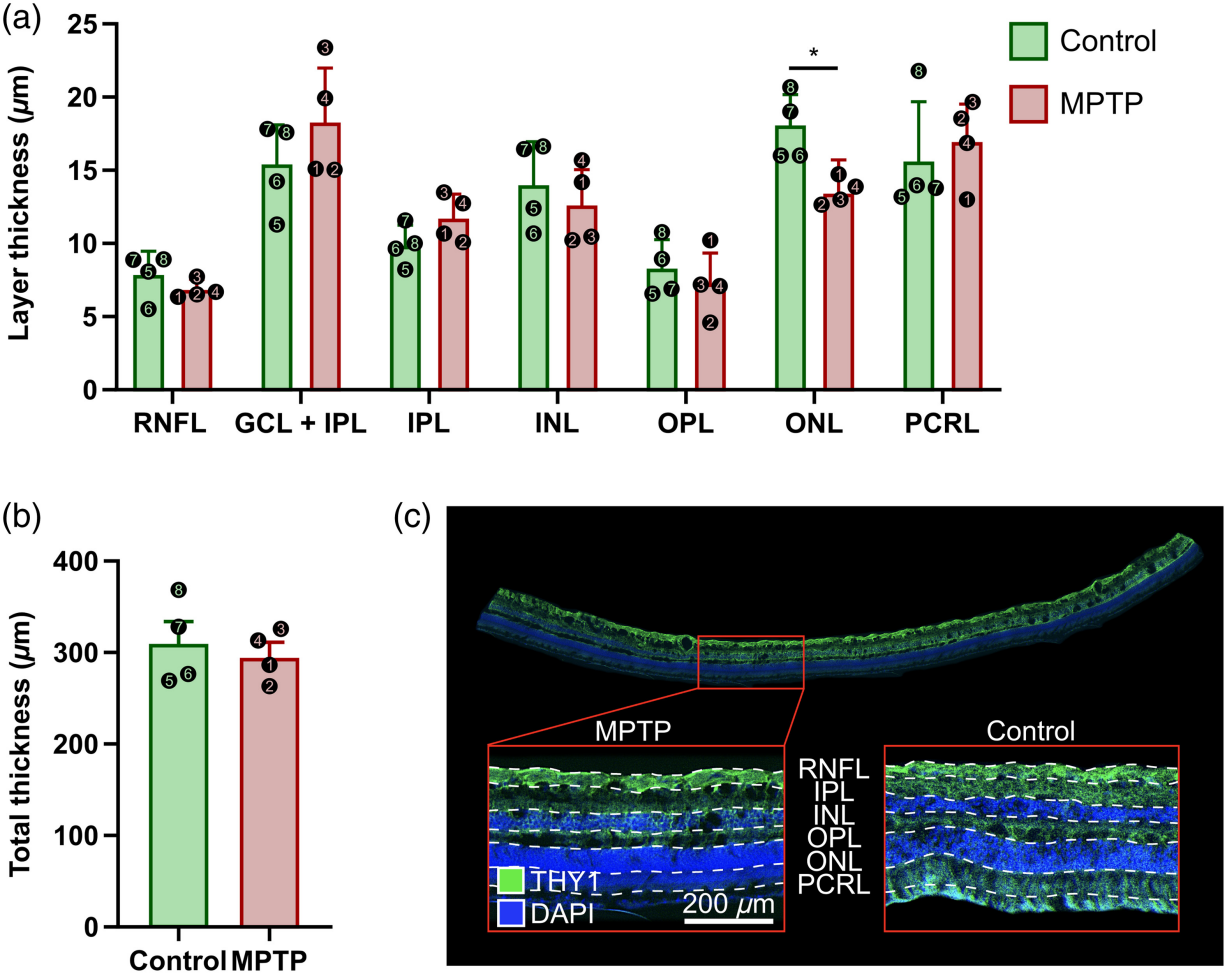
Post-mortem analysis comparing the thickness of each retinal layer (a) and the total retinal thickness (b) between the control and MPTP groups. The numbered dots represent the NHP numbers, as indicated in Table 1. The bars represent the mean and SD of the four NHPs. *P<0.05. (c) Fluorescent microscopy image of the cross-sectional retina illustrating the delineation of the individual layers.

To find if the pronounced changes we observed in spectral shape from the 480- to 525-nm band following MPTP administration correlated with morphological or cellular changes in the composition of the retina, the average thicknesses of each layer of the retina were calculated for each animal that received MPTP and compared with control animals that did not receive MPTP. The data were then averaged across all four animals of each condition. The RNFL showed a −12.88% difference in thickness in MPTP animals when compared with control animals (15.77±3.17  μm for control versus 13.74±1.40  μm for MPTP), the GCL + IPL showed a difference of +18.40% (30.90±5.31  μm for control versus 36.59±7.41  μm for MPTP), the IPL showed a difference of +18.88% (19.76±2.72  μm for control versus 23.49±3.25  μm for MPTP), the INL showed a difference of −9.90% (28.03±5.88  μm for control versus 25.26±4.83  μm for MPTP), the OPL showed a difference of −12.16% (16.65±3.87  μm for control versus 14.63±4.05  μm for MPTP), the ONL showed a statistically significant difference of −25.79% (36.21±4.14  μm for control versus 26.87±4.54  μm for MPTP) (P=0.029), and the PRCL showed difference of +8.45% (31.29±8.08  μm for control versus 33.93±5.13  μm for MPTP). The thickness of the entire retina showed a −7.40% difference in MPTP animals compared with control animals (155.31±23.34  μm for control versus 143.82±12.50  μm for MPTP) (Fig. 5).

## 4 Discussion

The goal of this study was to identify alterations to DRS in PD using an NHP model to find potential biomarkers and to assess if any cellular changes of the retina could correlate with differences observed in spectral measurements. Spectra were recorded in the ONH and the retina temporal to the ONH. Using these data, blood oxygen saturation was quantified, and the spectral shapes were analyzed in each region. MPTP administration resulted in no consistent changes to blood oxygen saturation. However, consistent changes were observed in the spectral shapes within both ROIs, especially to the slope of the spectra below 525 nm. Because the optical properties of a tissue are dependent on its physical structure and composition, these unique results provide evidence that MPTP alters these properties of the retina.

Blood oxygen saturation was unaffected by MPTP. Within the ONH, changes were negligible, and when averaged, none were statistically significant. Interestingly, within the retinal tissue, all animals had a slight increase in blood oxygen saturation [Fig. 3(b)]. Based on our results, we cannot conclude that MPTP has an effect on blood oxygen saturation. However, with a larger cohort, the increases observed in the retinal tissue could remain consistent and lead to a statistically significant difference.

It is yet unclear exactly if MPTP or PD may affect blood oxygen saturation. Some research has been conducted in this regard, but the evidence is preliminary. Valente et al.^32^ used a digital pulse oximeter to measure the peripheral blood oxygen saturation of individuals with PD and controls during an active tilt test. They found a significant increase in blood oxygen saturation in the PD group compared with the control group, although the difference was small (98% versus 96%). As for retinal oximetry, there is currently no reported data using this technique in PD patients. However, it has been performed in other neurological conditions, and the successfully recorded differences in blood oxygen saturation in the eye, specifically arterio-venal differences, were obtained. Einarsdottir et al.^22^^,^^23^ published two papers on AD and MS. The results indicate that, in AD individuals, retinal oxygen saturation was higher in both arterioles and venules, whereas in MS individuals, it was only higher in venules with no differences in arterioles. It is thought that such results could be caused by impaired oxygen metabolism in these diseases. Given that mitochondrial dysfunction and vascular changes have been observed in PD, it could be expected that oxygen metabolism could also be affected.^21^^,^^24^^,^^25^ Similarly, the current results found that all animals showed an increase of the StO2 in the retinal tissue, and although the increases were small, they were in the same order of magnitude than those observed in the studies mentioned above. It is also important to note that we used the MPTP model of PD that does not perfectly mimic every pathophysiological feature of PD. As such, more pronounced blood oxygen saturation differences might be observed in PD patients. Therefore, although investigation into how oxygen saturation is affected by PD is lacking, the present study in NHPs provides some insight into the potential for using blood oxygen saturation as a noninvasively recorded biomarker for neurodegenerative diseases.

When comparing the spectra between the pre- and postMPTP conditions, some consistent changes were observed across all animals. For both ROIs, the calculated slopes were greatly reduced, especially for the retinal tissue. This remained consistent while looking at the mean slope of individual animals, with only MPTP2 showing a slight increase in the retinal tissue. Furthermore, this phenomenon was also observed in almost every individual eye under most conditions for which data were available, with only the right eye of MPTP2 showing an increased slope postMPTP (Tables S6 and S7 in the Supplementary Material). This further illustrates the consistency of the effect of MPTP on the spectra slopes.

Typically, a change to the optical properties of the tissue is imparted by a change to the cellular or chemical composition of the tissue itself. Some research has been done to determine the optical properties of different ocular components.^33^ However, it is still difficult to discern exactly which component has caused the observed changes to the spectra. The main contributors to light absorption are blood (in the form of oxygenated and deoxygenated hemoglobin) and ocular melanin.^34^ Blood volume fraction (BVF) is a metric that can be taken from the blood component of the spectrum. Many studies have found microvascular alterations (and therefore alterations to BVF) in the retina of PD patients using optical coherence tomography angiography.^35^^–^^39^ Although this has not yet been investigated in animal models of PD, changes to BVF could be an explanation for spectra alterations.

Spectra from the retinal tissue could have clearly been influenced by the presence of ocular melanin, a major absorber of the light that was emitted by the device. This contrasts with the ONH that is devoid of ocular melanin and largely consists of axonal fibers and blood vessels. Melanin absorbs more light at the lower wavelengths with decreasing influence as wavelengths become higher.^33^ Because we see a greater difference in slope in the retinal tissue below 525 nm compared with the ONH (Fig. 4), it is possible that this was caused by a change in ocular melanin. Unfortunately, it was not possible in the present study to make an accurate measurement of the quantity of ocular melanin to confirm if this was the case.

The post-mortem measurement of the retinal layers between control and MPTP-intoxicated NHPs showed a statistically significant reduction in the thickness of the ONL. Other observed differences included reduced thicknesses for the RNFL, INL, and OPL and increased thicknesses for the GCL, IPL, and PRCL. Furthermore, the overall thickness of the retina was thinner in MPTP NHPs. The significant thinning of the ONL provides evidence that MPTP can cause morphological changes to the retina, which would alter its optical properties, thereby providing an explanation for the changes observed in the recorded spectra.

Morphological changes caused by MPTP may reflect those observed in PD. Indeed, many studies have found thinning of retinal layers, particularly the RNFL and the GCL + IPL, in PD patients when compared with controls using optical coherence tomography (OCT), an imaging technique that noninvasively constructs cross-sectional images of tissue. A meta-analysis published by Chrysou et al.^12^ compiled data from all suitable research between 2011 and 2018 comparing the thickness of retinal layers between PD subjects (1916 total) and non-parkinsonian control subjects (2006 total). From the compiled data, there was a significant thinning of the RNFL and the GCL + IPL in PD subjects [overall effect: −4.2  μm (99.3  μm versus 94.8  μm) and −3.6  μm (81.7  μm versus 78.1  μm), respectively]. Other layers showed no significant difference in thickness. Interestingly, only two studies reported the thickness of the ONL, which both showed a reduction in thickness of −2.9  μm (124.1 versus 121.2) and −9.2  μm (68.8 versus 59.6), respectively. These results show some similarities and differences to ours. First, our results showed a thinning of the RNFL, similar to what was presented by the meta-analysis, with an even greater effect size. However, although we found that the IPL had increased in thickness, most of the studies appeared to show a decrease in thickness in PD patients. Furthermore, although we found consistent decreases in thicknesses of the INL and OPL, this was less consistently observed in the previous PD studies. The statistically significant reduction in the ONL thickness that we observed is in line with the results obtained by the two studies from the meta-analysis, which both showed notable decreases. It is clear from these results that, in the future, more attention should be put on the ONL when measuring retinal layer thicknesses in PD patients using OCT as it appears to be a layer that is affected by PD but has so far been underreported.

Fewer animal studies have been conducted looking into the effect of MPTP on the retina. Schneider et al.^40^ used OCT to measure thicknesses of the RNFL, fovea thickness, and macula volumes in normal and MPTP cynomolgus monkeys. The average thinning of the RNFL was statistically significant in the MPTP-intoxicated monkeys, specifically in the inferior and nasal quadrants. The mean foveal thickness and macula volume were also significantly reduced. This provides evidence that MPTP in monkeys is not only effective at mimicking pathophysiological hallmarks of PD in the brain but also in the retina.

Although the significant reduction in ONL thickness could be an explanation for the change in spectra slope from the retinal tissue, this does not explain the change from the ONH. Unlike the retina, the ONH does not contain the different retinal layers and only consists of ganglion cell axons and blood vessels. Previous studies analyzing the retina in PD or MPTP animals typically found a reduction in the RNFL thickness, the ganglion cell axonal fibers that relay to the ONH, indicating a reduction in the density of ganglion cells/axonal fibers that should influence the physical composition of the ONH and therefore its optical properties.^12^^,^^40^ Hence, although the thinning of the RNFL in the present study was not found to be statistically significant, the observed change in spectra slope within the ONH indicates that thinning of the RNFL may have happened.

One challenge with using NHPs as experimental subjects is their availability. Not only is it difficult to acquire them and expensive to house and care for them, but ethical limitations also ensure that as few animals are used as possible. This meant that there was limited availability of subjects for both the *in vivo* tests, reducing the statistical power, and for control subjects needed for the post-mortem comparisons. For this reason, the control subjects that we could acquire did not match for sex and age. Therefore, it is difficult to discern if the post-mortem differences were caused by MPTP or if these confounding variables may have had an influence. A larger sample size would be needed to assess any sex or age-related differences. Denk et al.^41^ found sex differences in the retinal thickness of cynomolgus macaque monkeys, potentially explaining the difference observed in the present study. Regarding the difference in age of the animals, retinal development is largely complete by birth, with only rearrangement and elongation of photoreceptors occurring postnatally and primarily in the fovea, whereas cells in the perifovea, from which measurements were taken, are largely unchanged.^42^^,^^43^ Furthermore, macaque retinal cell redistribution has been found to reach full maturity by 15 months.^44^^,^^45^ By comparison, thinning of the macaque RNFL, thought to represent overall retinal thinning, has been found to occur at a rate of 0.70  μm per year from birth, illustrating that age-related thinning between the groups should be negligible, particularly for the difference seen in the ONL.^46^ Therefore, it is unlikely that the age difference would have had a major effect on differences observed in retinal thickness.

To our knowledge, this is the first study to investigate changes in the ocular blood oxygen saturation caused by MPTP intoxication in NHPs. Previously, only one other device has been used to noninvasively measure retinal oximetry, the Oxymap T1 (Oxymap ehf, Reykjavik, Iceland). The Oxymap T1 focuses on recording oximetry by comparing the optical densities of two images taken at two wavelengths: 570 nm (the reference wavelength unaffected by oxygenated hemoglobin) and 600 nm (a wavelength affected by oxygenated hemoglobin). This is performed only in large observable blood vessels through an automatic detection performed by the device software. The present study presents some of the first analyses of the total raw spectrum recorded from the retina. Data were obtainable from any part of the eye fundus, not just the blood vessels, allowing for selective acquisition and comparison between specific regions such as the ONH and the retina. Having the raw spectrum also allowed for the analysis of spectral components other than blood oxygen saturation, such as those affected by the physical structure of the retina.

These results provide a foundation for future work. Currently, PD diagnosis can only happen after the presentation of motor symptoms, during which around 50% of dopaminergic neurodegeneration in the SNc has occurred.^8^ If earlier diagnosis were possible, it would provide the opportunity to try to prevent further degeneration and slow the progression of the disease. It is known that vision difficulties can manifest in individuals prior to the official PD diagnosis; therefore, it is likely that changes in the eye fundus are occurring before motor symptoms.^47^ To investigate if this is the case, a similar study could be conducted with a controlled dosage of MPTP to mimic the prodromal stage of the disease, during which similar recordings would be made to establish if similar results to the present study can be obtained. Similar experiments could also be conducted in other animal models of the disease, such as vector-mediated models causing cellular accumulation of α-synuclein. Finally, to confirm that these results appear in PD, recordings would need to be made in PD patients and compared with matched healthy controls to see if the differences between the two groups will be the same as the changes seen in the MPTP-intoxicated monkeys.

## Supplementary Material

10.1117/1.NPh.12.2.025008.s01

## Data Availability

All data in support of the findings of this paper are available within the article or as Supplementary Material.
