# Supplementary material for: Retinal changes detected by diffuse reflectance spectroscopy in parkinsonian monkeys
Source: Neurophotonics. 2025 May 5;12(2):025008. doi: 10.1117/1.NPh.12.2.025008 (PMC12052396; doi:10.1117/1.NPh.12.2.025008)
Supplement: Supplementary file 1 [file NPh_012_025008_SD001.docx]

*Supplementary table 1: Mean blood oxygen saturation (%) from the ONH of the left and right eyes of each animal between Pre and PostMPTP conditions. OS = left eye, OD = right eye.*

| **Animal** | **Eye** | **Mean saturation PreMPTP** | **Mean saturation PostMPTP** |
| --- | --- | --- | --- |
| **MPTP1** | OS | 43.19 | 46.00 |
|  | OD | No data | No data |
| **MPTP2** | OS | 48.48 | 44.87 |
|  | OD | 46.27 | 48.97 |
| **MPTP3** | OS | 50.50 | 49.27 |
|  | OD | 47.92 | 47.75 |
| **MPTP4** | OS | 45.17 | 46.60 |
|  | OD | 46.75 | 47.69 |

*Supplementary table 2: Mean blood oxygen saturation (%) from the ONH during each recording session between the left and right eyes of each animal. OS = left eye, OD = right eye.*

| **Animal** | **Recording session** | **Mean saturation OS** | **Mean saturation OD** |
| --- | --- | --- | --- |
| **MPTP1** | PreMPTP | 43.19 | No data |
|  | PostMPTP 1 | No data | No data |
|  | PostMPTP 2 | 46.00 | No data |
|  | PostMPTP 3 | No data | No data |
| **MPTP2** | PreMPTP | 48.48 | 46.27 |
|  | PostMPTP 1 | 44.45 | 47.85 |
|  | PostMPTP 2 | 45.83 | 50.01 |
|  | PostMPTP 3 | 43.94 | No data |
| **MPTP3** | PreMPTP | 50.50 | 47.92 |
|  | PostMPTP 1 | 49.43 | 50.02 |
|  | PostMPTP 2 | No data | No data |
|  | PostMPTP 3 | 49.18 | 46.95 |
| **MPTP4** | PreMPTP | 45.17 | 46.75 |
|  | PostMPTP 1 | No data | No data |
|  | PostMPTP 2 | 47.27 | No data |
|  | PostMPTP 3 | 46.08 | 47.69 |

*Supplementary table 3: Mean blood oxygen saturation (%) from the nasal retina of the left and right eyes of each animal between Pre and PostMPTP conditions. OS = left eye, OD = right eye.*

| **Animal** | **Eye** | **Mean saturation PreMPTP** | **Mean saturation PostMPTP** |
| --- | --- | --- | --- |
| **MPTP1** | OS | 37.97 | 39.12 |
|  | OD | 40.91 | 40.78 |
| **MPTP2** | OS | No data | 39.46 |
|  | OD | 37.48 | 38.45 |
| **MPTP3** | OS | 38.83 | 39.80 |
|  | OD | 37.34 | 39.88 |
| **MPTP4** | OS | 37.98 | 37.95 |
|  | OD | 37.00 | 38.17 |

*Supplementary table 4: Mean blood oxygen saturation (%) from the nasal retina during each recording session between the left and right eyes of each animal. OS = left eye, OD = right eye.*

| **Animal** | **Recording session** | **Mean saturation OS** | **Mean saturation OD** |
| --- | --- | --- | --- |
| **MPTP1** | PreMPTP | 37.97 | 40.91 |
|  | PostMPTP 1 | No data | 40.78 |
|  | PostMPTP 2 | No data | No data |
|  | PostMPTP 3 | 39.12 | No data |
| **MPTP2** | PreMPTP | No data | 37.48 |
|  | PostMPTP 1 | No data | 37.44 |
|  | PostMPTP 2 | 38.85 | 35.61 |
|  | PostMPTP 3 | 40.72 | 39.51 |
| **MPTP3** | PreMPTP | 38.83 | 37.34 |
|  | PostMPTP 1 | No data | 39.88 |
|  | PostMPTP 2 | No data | No data |
|  | PostMPTP 3 | 39.80 | No data |
| **MPTP4** | PreMPTP | 37.98 | 37.00 |
|  | PostMPTP 1 | 39.27 | No data |
|  | PostMPTP 2 | 36.95 | No data |
|  | PostMPTP 3 | No data | 38.17 |

*Supplementary table 5: Mean spectral slope (480 - 525 nm) from the ONH of the left and right eyes of each animal between Pre and PostMPTP conditions. OS = left eye, OD = right eye.*

| **Animal** | **Eye** | **Mean slope PreMPTP** | **Mean slope PostMPTP** |
| --- | --- | --- | --- |
| **MPTP1** | OS | -0.0070 | -0.0048 |
|  | OD | No data | No data |
| **MPTP2** | OS | -0.0059 | -0.0056 |
|  | OD | -0.0055 | -0.0050 |
| **MPTP3** | OS | -0.0052 | -0.0048 |
|  | OD | -0.0095 | -0.0048 |
| **MPTP4** | OS | -0.0047 | -0.0034 |
|  | OD | -0.0054 | No data |

*Supplementary table 6: Mean spectral slope (480 - 525 nm) from the ONH during each recording session between the left and right eyes of each animal. OS = left eye, OD = right eye.*

| **Animal** | **Recording session** | **Mean slope OS** | **Mean slope OD** |
| --- | --- | --- | --- |
| **MPTP1** | PreMPTP | -0.0070 | No data |
|  | PostMPTP 1 | No data | No data |
|  | PostMPTP 2 | -0.0048 | No data |
|  | PostMPTP 3 | No data | No data |
| **MPTP2** | PreMPTP | -0.0059 | -0.0055 |
|  | PostMPTP 1 | -0.0041 | -0.0049 |
|  | PostMPTP 2 | -0.0052 | -0.0051 |
|  | PostMPTP 3 | -0.0069 | -0.0054 |
| **MPTP3** | PreMPTP | -0.0052 | -0.0095 |
|  | PostMPTP 1 | -0.0055 | -0.0042 |
|  | PostMPTP 2 | No data | No data |
|  | PostMPTP 3 | -0.0044 | -0.0050 |
| **MPTP4** | PreMPTP | -0.0047 | No data |
|  | PostMPTP 1 | No data | No data |
|  | PostMPTP 2 | -0.0034 | No data |
|  | PostMPTP 3 | No data | No data |

*Supplementary table 7: Mean spectral slope (480 - 525 nm) from the nasal retina of the left and right eyes of each animal between Pre and PostMPTP conditions. OS = left eye, OD = right eye.*

| **Animal** | **Eye** | **Mean slope PreMPTP** | **Mean slope PostMPTP** |
| --- | --- | --- | --- |
| **MPTP1** | OS | -0.0084 | -0.0080 |
|  | OD | -0.0050 | -0.0015 |
| **MPTP2** | OS | No data | -0.0040 |
|  | OD | -0.0054 | -0.0077 |
| **MPTP3** | OS | -0.0033 | -0.0034 |
|  | OD | -0.0063 | -0.0040 |
| **MPTP4** | OS | -0.0063 | -0.0048 |
|  | OD | -0.0092 | No data |

*Supplementary table 8: Mean spectral slope (480 - 525 nm) from the nasal retina during each recording session between the left and right eyes of each animal. OS = left eye, OD = right eye.*

| **Animal** | **Recording session** | **Mean slope OS** | **Mean slope OD** |
| --- | --- | --- | --- |
| **MPTP1** | PreMPTP | -0.0084 | -0.0050 |
|  | PostMPTP 1 | No data | -0.0015 |
|  | PostMPTP 2 | No data | No data |
|  | PostMPTP 3 | -0.0080 | No data |
| **MPTP2** | PreMPTP | No data | -0.0054 |
|  | PostMPTP 1 | No data | -0.0059 |
|  | PostMPTP 2 | -0.0035 | -0.0086 |
|  | PostMPTP 3 | -0.0049 | -0.0079 |
| **MPTP3** | PreMPTP | -0.0033 | -0.0063 |
|  | PostMPTP 1 | No data | -0.0040 |
|  | PostMPTP 2 | No data | No data |
|  | PostMPTP 3 | -0.0034 | No data |
| **MPTP4** | PreMPTP | -0.0063 | -0.0092 |
|  | PostMPTP 1 | -0.0041 | No data |
|  | PostMPTP 2 | -0.0054 | No data |
|  | PostMPTP 3 | No data | No data |
